# PROTOCOL: An evidence and gap map of studies of implementation issues for interventions for those affected by and at risk of homelessness in high‐income countries

**DOI:** 10.1002/cl2.1305

**Published:** 2023-02-21

**Authors:** Sabina Singh, Suzanne Fitzpatrick, Monisha Lakshminarayanan, Ashrita Saran, Jenny Wood, Ting Yang, Howard White

**Affiliations:** ^1^ Campbell South Asia Global Development Network New Delhi India; ^2^ Institute for Social Policy, Housing and Equalities Research (I‐SPHERE) Heriott‐Watt University Edinburgh UK; ^3^ Campbell Collaboration Beijing China; ^4^ Global Development Network New Delhi India

## Abstract

This is the protocol for a Campbell systematic review. The objectives are as follows. The proposed evidence and gap map will present relevant process evaluations and other studies of barriers and facilitators, both qualitative and quantitative, for eligible homelessness interventions to highlight the issues arising in the implementation of these interventions. Specifically, the objectives of the map are to: (i) develop a clear taxonomy of interventions and implementation issues (e.g., barriers and facilitators—factors which works as barriers to hinder successful implementation of policies and programmes and factors which facilitate the intervention and therefore support its implementation) related to homelessness in high‐income countries; (ii) map available systematic reviews and primary studies of the implementation issues of interventions for those experiencing homelessness and those at risk of homelessness, with an overview provided in a summary report; (iii) provide a searchable database of included studies accessible to research users via CHI website.

## 1 BACKGROUND

### 1.1 The problem, condition or issue

Homelessness remains a major societal challenge in high‐income countries as it affects a large number of people. In the United States alone, over half a million people are affected by homelessness. The number of households in England and Scotland that approached local authorities for statutory assistance in 2019‐20 was nearly 288,000 and 18,465 households respectively^1^.

The challenge of homelessness is not reflected in the above‐mentioned numbers alone. There are many people who live in precarious or unsafe situations. Also, those living in unstable housing or at risk of homelessness add to the challenge. As per the Shelter estimates, as many as 320,000 people may be experiencing homelessness across Britain at the end of 2018 (Reynolds, 2018). The number of households experiencing the most acute forms of homelessness including sofa surfing, rough sleeping, and sleeping in tents and cars, as estimated by Crisis was close to 160,000 (Bramley, 2017).

Homelessness is also a major challenge in the developing countries. However, the causes and the interventions to address homelessness in developing countries are qualitatively different from that in the developed countries. Speak (2004) suggests that the complexity of homelessness in developing countries be understood from the context‐specific manifestation of homelessness rather than juxtaposing the typologies of homelessness for developed countries. The context and causes not only vary among the developed and developing countries but also within the developing countries.

Homelessness, even of short durations, can result in socioeconomic exclusion with reduced access to a range of social services and reduced employment possibilities. People experiencing homelessness have worse health outcomes, and there is a mutual relationship between homelessness and other social disadvantages such as mental health problems and substance abuse. The social costs and consequences of homelessness are substantial. People affected by homelessness die at a much younger age than the general population (Thomas, 2012). Spending to reduce homelessness has been estimated to save the public purse close to £20,000 per homeless person (PWC, 2018).

Effective interventions are therefore required to place and keep people in stable housing, and address the health and wider support needs of all people experiencing or at risk of homelessness. There is a range of interventions to try to prevent homelessness and to increase housing stability. In order to inform policy, it is important to know both what works and how to make interventions work in different contexts. As a basis for making this assessment, the UK Centre for Homelessness Impact (CHI) has commissioned two evidence and gap maps (EGMs) to survey the evidence base. The first is on effectiveness, for which the protocol has been published as White et al. (2019). The second map is on implementation issues, for which this is the protocol.

Following the effectiveness map, which can guide a decision‐maker to identify an appropriate intervention for their client group, this implementation issues map allows the decision‐maker to focus on evidence about the factors critical to effective implementation of the intervention. This implementation issues EGM is the first map of qualitative studies applying this approach. It is thus methodologically innovative. It endeavours to cover relevant process evaluations and other studies of barriers and facilitators for eligible homelessness interventions with a focus on implementation issues. The terms barriers and facilitators are used synonymously.

Development of the map will support efforts to tackle socioeconomic exclusion, and sustained deprivation and inequality. It will support related research initiatives such as Inclusion Health (Luchenski et al., 2018). And importantly the maps will support a suite of evidence tools produced by the CHI (homelessnessimpact.org).

### 1.2 Scope of the EGM

*Full name*: An EGM of Studies of the Implementation Issues for Interventions for Those Affected by and at Risk of Homelessness in High‐Income Countries.

*Short name*: Homelessness: An EGM (Implementation issues).

The European Typology of Homelessness and Housing Exclusion (ETHOS) defines a person as homeless if they have a deficit in one or more of the physical, legal, and social domains—also described as being roofless or houseless (FEANTSA, 2012, p. 11). We have, thus, broadly defined homelessness so as to include both those experiencing homelessness—that is, those who have no accommodation and so sleep on the street (sleeping rough) and those in temporary (i.e., transitional), insecure or poor‐quality housing (European Commission, no date). People in temporary shelters or other transitional accommodation are still considered homeless. Those at risk of homelessness may currently be in satisfactory accommodation but at risk of losing it—for example, because of loss of employment or other income source. Thus, our broader definition of homelessness includes not only people who are currently homeless but also those at risk of homelessness or those who need assistance to protect them from being homeless again.

The interventions, which are listed below, are interventions whose main purpose is to improve the welfare of those experiencing or at risk of homelessness in high income countries, primarily through legal provisions that facilitate people's access and rights to house, health and social care; education, employment and other preventive measures for homelessness, to name a few. The welfare corresponds to the health and well‐being needs of the homeless, such as access to safe housing, education, employment and healthcare services. The barriers and facilitators were identified through an iterative process as mentioned below.

### 1.3 Conceptual framework of the EGM

**Figure 1:**
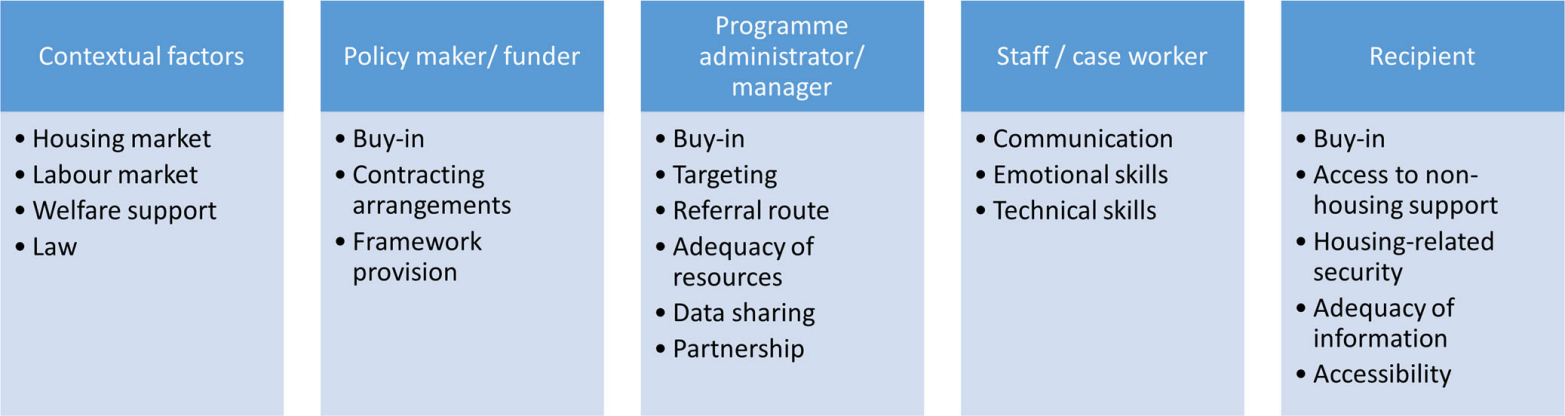
Implementation issues framework.

Figure 1 shows the implementation issues framework used for the map. The implementation science framework by Aarons et al. (2011) was taken as the base to identify initial implementation issues categories. These categories were further developed in two stages using a grounded theory approach whereby the identified categories were piloted against eligible studies first by Campbell Collaboration team and Queen's University Belfast, and then by the Heriot‐Watt University research team.

The framework identifies different groups involved in any intervention, as well as contextual factors. For each of these, we identified the factors which may be either barriers or facilitators affecting implementation which matter for that group. These barriers and facilitators can be thought of as the assumptions of the things which need to be in place for programmes to work effectively. For our purpose, barriers were any factors that posed hindrance or challenges to the successful implementation of the programme/intervention while facilitators were the factors that enabled the successful implementation of the programme/intervention. We have thus used broader definitions of barriers and facilitators and these operational definitions thus suggest that barriers and facilitators could be synonymised and may turn out to be either of the one or both as per the context.

The intervention categories in this map are same as in the effectiveness map (White et al., 2019). The intervention categories for that map were also identified through an iterative process. The first set of categories were based on those used in a systematic review of interventions to improve housing stability (Munthe‐Kaas et al., 2018). A network of homelessness researchers in the United Kingdom contributed in revising these categories substantially. The revised categories were piloted against a set of eligible studies resulting in further revisions by the Campbell Collaboration research team. The intervention categories were further revised first through stakeholder consultation workshop and then subsequently by CHI based on user feedback.

### 1.4 Why it is important to develop the EGM

Currently, there is no single resource which allows policy makers, practitioners and researchers working to improve the welfare of those experiencing homelessness to access the available relevant evidence on which programmes work. The review team is working with the UK CHI to develop the evidence architecture for the sector.

The CHI is establishing itself as a ‘one stop shop’ for evidence for policy makers and practitioners in the sector. As a first step, working with the Campbell Collaboration, the Centre is producing to two evidence maps of evidence on homelessness. This protocol is for the map of implementation studies of interventions to improve the Welfare of those Experiencing Homelessness. The other map shows studies of effectiveness for such interventions as identified in impact evaluations and systematic reviews of those studies. The two maps together will comprise the largest single source globally of evidence on interventions for those experiencing and at risk of homelessness.

CHI aims to improve the welfare of people affected by homelessness by providing evidence‐based resources for policy makers and practitioners. The EGMs are the first part of that evidence architecture, and a building block for what will come next. The maps will identify the evidence to be used in the Centre's online evidence resources. And the maps will inform the future policy‐oriented research programme of the Centre.

### 1.5 Existing EGMs

The only EGM in relation to homelessness is the one on effectiveness (White et al., 2019) which has a different focus, and so different included studies to this map.

There are several reviews on implementation issues but the scope is not as broad as this map. There are, however, systematic reviews on implementation issues of interventions for homeless such as a systematic review on acceptability of health and social interventions for persons with lived experience of homelessness (Magwood et al., 2019). Another review details the factors affecting the effectiveness of Housing First (Chambers et al., 2018).

## 2 OBJECTIVES

The proposed EGM will present relevant process evaluations and other studies of barriers and facilitators, both qualitative and quantitative, for eligible homelessness interventions to highlight the issues arising in the implementation of these interventions. Specifically, the objectives of the map are to:

(i) develop a clear taxonomy of interventions and implementation issues (e.g., barriers and facilitators—factors which works as barriers to hinder successful implementation of policies and programmes and factors which facilitate the intervention and therefore support its implementation) related to homelessness in high‐income countries.
(ii) map available systematic reviews and primary studies of the implementation issues of interventions for those experiencing homelessness and those at risk of homelessness, with an overview provided in a summary report.
(iii) provide a searchable database of included studies accessible to research users via CHI website.

## 3 METHODS

### 3.1 Defining EGMs

An EGMs for a specific research question depicts the available evidence that is synthesized systematically following systematic search, screening, data extraction and critical appraisal of included studies. EGMs are, thus, systematic evidence synthesis products. EGMs are useful to identify evidence gaps for a particular research question. They prove useful for decision‐makers, researchers and research commissioners alike by making available the links to relevant studies and thereby increasing the discoverability and use of studies. EGMs can also be used to generate higher‐level evidence products such as guidelines (White et al., 2020).

This EGM is an implementation issues map in which the primary dimensions are the rows and columns of the map which are, respectively, intervention categories (and sub‐categories) and implementation issues (and sub‐domains). Secondary dimensions, such as country and target group will be included as filters.

#### 3.1.1 EGM framework

The EGM framework will inform the inclusion and exclusion criteria of the EGM. Here we describe the population, intervention, barriers and facilitators and study designs (PIOS) for the map.

##### Population

The population is individuals and families who are homeless or at risk of becoming homeless.

Population sub‐groups of interest are listed under filters.

##### Intervention

The intervention categories are the same as those used for the effectiveness map. Interventions are broadly defined to include legislation and policies which are intended to improve the housing status of individuals and families, as well as prevention approaches. The complete list of interventions is: (1) legislation, (2) prevention, (3) services and outreach, (4) accommodation‐based interventions, (5) employment, (6) health and social care, (7) education and skills, (8) communication, and (9) financing.

Table 1 lists the intervention sub‐categories, with definitions.

**Table 1:** Intervention categories and sub‐categories.

Legislation	Housing/Homelessness Legislation	Legislation pertaining to availability of/access to housing, or the rights of those experiencing homelessness
	Welfare Benefits	Legislation for welfare programmes to help people experiencing homelessness, or to help prevent people who are at risk of becoming homeless from losing their home.
	Health and social care	Legislation for access to health and social care to help people experiencing homelessness, or to help people who are at risk of becoming homeless.
Prevention	Welfare and Housing Support	State contribution towards housing costs and other welfare payments and services, whether directly made to tenants or indirectly paid to service provider (e.g., landlords—examples in the UK: Local Housing Alliance, Universal Credit, etc; US: vouchers) from the state or non‐state actors. This includes other welfare benefits such as childcare if studied in the context of homelessness.
	Housing supply	Policies promoting the development of new housing supply that is affordable and accessible (whether for social or private purposes)—this includes the construction, conversion of homes, and re‐purposing. Interventions comprise changes to legislation, financing mechanisms and other support for developers and those conditioning units for these purposes.
	Family therapy and mediation	Counselling and mediation of conflicts, usually between young people and their family so they may avoid becoming homeless or reduce other risky behaviour. (Landlord‐tenant mediation is a separate category)
	Landlord‐tenant mediation	Mediation between landlords and tenants to encourage landlords to accept tenants with history of homelessness, substance abuse etc. and to address conflicts. This may include, but is not limited to mediation around arrears, noise and substance abuse, damage to property, eviction, etc.
	Discharge interventions	Provision of services, including accommodation, to people being discharged from institutions (care, hospitals, prison, armed forces) to avoid people being discharged into homelessness. This may include coordination between agencies, accomodation, and other services tailored to their needs. It refers to both interventions whilst in the insitution and community‐based interventions focused on recently discharged persons.
Services and outreach	Feeding including soup runs	Provision of food in street and day centre settings to people experiencing homelessness.
	In‐kind support (exc. food)	Provision of clothing, hygiene products, household items etc., but excluding food
	Day centres	Centres open only during the day to provide food and services for people experiencing homelessness. This code is used if the day centre itself is being evaluated in the study rather than being the setting for the intervention.
	Outreach	Outreach refers to work with people sleeping rough or in temporary or unstable accommodation. Outreach workers go out, including late at night and in the early hours of the morning, to locate people who are rough sleeping or work with day centres, shelters etc. The role of outreach teams varies but usually outreach workers seek to engage with people and check their immediate health and wellbeing, collect basic information about their situation, facilitate access to emergency accommodation or other accommodation (such as hostels or Housing First), and inform them about day centres and other services they might have available. Outreach models vary and may include enforcement (e.g., police officials) to remove people from the streets or enforce specific behaviours.
	Reconnection of rough sleepers	Reconnecting people experiencing homelessness (rough sleepers) or at risk of homelessness (e.g., those discharged from institutions such as prison) to their ‘home’ location (usually another city, state or country where they have networks, access to services, etc) by providing the cost of transport for relocation.
	Psychologically informed environments.	Psychologically informed environments are interventions designed to take into account the psychological profile of the client. Community Reinforcement Approach (CRA) is included here.
	Case management (inc. Critical Time Intervention)	Individual‐level approach to ensure coordination of services. The case worker (can be social worker or dedicated case worker from another agency) works directly with the client to ensure that the client has access to all applicable services, for example, health, training and social activities. A specific application of the case work approach is critical time intervention (CTI) which provides a person (or family) in transition between types of accommodation and at risk of homelessness with a period of intensive support from a caseworker. The caseworker will have established a relationship with the client before the transition—for example, before discharge from hospital or prison. Critical time intervention involves three stages: (1) direct support to the client and assessing what resources exist to support them, (2) trying out and adjusting the systems of support as necessary, and (3) completing the transfer of care to existing community resources.
	Service coodination, co‐location or embedded in mainstream services	System‐based approaches to ensuring coordination of service delivery. Coordination may refer to ensuring communication between relevant services. Coordination also includes providing services in the same location or adjacent to mainstream services. Co‐location refers to multiple services being available in the same physical location (e.g., housing and job search services in the same location). Embedded refers to services being integrated in the same place (e.g., housing and other services within a hospital context). A specific example is coordinated assessment. Refers to case workers making broad assessments of people at risk as homelessness on different factors that affect their risk. Try to ensure different services employ the same assessment tools to standardize practice.
	Veterinary services	Access to veterinary services for pets of people experiencing homelessness
	Legal advice	Legal assistance and advice delivered away from primary service/office to the homeless population.
Accommodation‐based services	Shelters	Homeless shelters are a basic form of temporary accommodation where a bed is provided in a shared space overnight. One of the key features of a homeless shelter is that it is transitional and an option for those homeless who are not yet eligible for more stable accommodation. Shelters are not usually seen as stable forms of accommodation as the individual must vacate the space during daytime hours with their belongings. One of the key differences with hostels is the need to vacate the premises during the day.
	Hostels	Hostels for homeless people are designed provide short‐term accommodation, usually for up to two years depending on available move‐on accommodation. Typically shared accommodation projects with individual rooms and shared facilities including bathrooms and kitchens. Hostels have staff on site 24 h a day and during the daytime provide support to residents on issues including welfare benefits and planning their move from the hostel into more medium to long‐term accommodation.
	Temporary accommodation	Temporary accommodation includes a range of housing options which are more stable than shelters or hostels, such as transitional housing and residential programmes.
	Host homes	Emergency Host homes are emergency short‐term placements in volunteers’ own homes in the community for people who are homeless or at risk of homelessness. Hosting services are often aimed at young people with low support needs, but exist for other groups too, such as people who have been refused asylum.
	Rapid Rehousing	Rapid rehousing places those who experiencing homelessness into accommodation as soon as possible. The intervention provides assistance in finding accommodation, and limited duration case work to connect the client to other services.
	Housing First	Housing First offers accommodation to homeless people with multiple and complex needs with minimal obligations or conditions being placed upon the participant. Housing First provides safe and stable housing to all individuals, regardless of criminal background, mental instability, substance abuse, or income.
	Social housing (with or without support)	Housing that is provided in the social sector. It may sometimes be provided alongside support services, this may be temporary or permanent. Examples of support that may be provided are health and money management (excluding Housing First and Rapid Rehousing). This is based on an institutional setting.
	Private Rental Sector (with and without support)	Housing that is provided in the private rental market where the tenant is fully responsible. This may or may not include additional support services as the focus is on the type of tenancy agreement (private).
	Continuum of Care	An approach to accommodation whereby people experiencing homelessness move through different forms of transitional accommodation until they are deemed ‘housing ready’ (e.g., stopped substance abuse) and allocated independent settled housing.
Employment	Mentoring, coaching and in‐work support	Mentoring and coaching to support job search including activities like practice interviews, review CVs, etc and on the job support for work performance.
	Flexible employment	Employment which can accommodate needs for the person experiencing homelessness.
	Vocational training and unpaid work experiences	Unpaid job placement or vocational training to provide work experience for people experiencing, or at risk of, homelessness.
	Paid work experiences	Paid job placement to provide work experience for people experiencing, or at risk of, homelessness.
Health and social care	Health services (physical and mental)	Providing direct access to, or facilitating access to, physical and mental health services for people experiencing homelessness.
	End of life care	End of life care for people experiencing or at risk of homelessness.
	Additional support	Services for people experiencing, or at risk of, homelessness who have substance misuse problems (including alcohol and other substances)
Education and skills	Life and social skills training	Life and social skill training including socio‐emotional skills, financial literacy (money management), tenancy management, and how to deal with ones home; for people experiencing or at risk of homelessness
	Mainstream education	General education at all levels for people experiencing, or at risk of, homelessness including children in families at risk of or experiencing homelessness.
	Homelessness awareness programmes in schools	School‐based programmes to raise awareness of homelessness [Not interventions to help school aged children attend school; these are under mainstream education).
	Recreational and creative activities	Recreational, social (e.g., social clubs) and creative (e.g., theatre) activities for people experiencing homelessness.
Communication	Advocacy campaigns	Campaigns by 3rd sector organizations which aim to improve awareness of the general public of homelessness, its causes, and its solutions, and promote rights of the homeless.
	Public information campaigns	Campaigns by government organizations which aim to improve awareness of the general public of homelessness, its causes, and its solutions, and promote rights of the homeless.
	Service availability	General communication activities to raise awareness amongst people experiencing homelessness, or at risk of homelessness, of the services available to them. Does not include case management, discharge etc which provides information or connects individuals to services.
Financing	Social Impact Bonds	Performance‐based financing for organizations commissioned to provide services to people experiencing homelessness. Not these are not interventions in themselves, but payment mechanisms for service deliverers.
	Direct financial support from public	Money given directly by individuals to those experiencing or at risk of homelessness

##### Outcomes (implementation issues)

In this map the column headings are not outcomes but implementation issues. The implementation issues categories (as shown in Table 2) were developed through an iterative process. An initial set of categories was developed by the Campbell Collaboration team based on the implementation science framework presented by Greg Aarons and colleagues (Aarons et al., 2011). These categories were assessed by researchers with expertise in homelessness and through piloting to arrive at the final list.

**Table 2:** Implementation issues categories.

Contextual factors	Housing market	Housing market conditions (quantity, quality, price)
	Labour market	Labour market conditions, such as amount and type of employment available, and factors affecting those who are homeless or having conditions correlated to homelessness.
	Welfare support	Factors related to welfare support (availability, type, value, timing) and restrictions.
	Law	Laws directly affecting people experiencing homelessness or at risk of homelessness.
Policy maker/funder	Buy‐in (Leadership, culture, priorities, commitment to programme)	The support of the leadership, organizational culture and incentives.
	Contracting arrangements with external agencies	Restrictions, incentives etc. arising from contractual arrangements.
	Framework provision (e.g., policies and guidelines)	Organizational policies, guidelines and requirements (formal or informal).
Programme administrator/manager/implementation agency	Buy in (Leadership, culture, priorities)	Understanding and support from programme staff and managers
	Identification of recipient/targeting mechanism	Process, rules, procedures, both de jure and de facto, used to identify programme beneficiaries
	Referral route (e.g., defined agency or contact)	Process, rules, procedures, both de jure and de facto, used to refer programme beneficiaries
	Sufficiency/Adequacy of Resources (space, time, staff, budget	Availability (quantity and quality) of resources of all kinds
	Alignment with existing protocol/procedures/guidelines	Whether a project or programme is well aligned with existing procedures etc.
	Monitoring data/Data sharing	Availability, collection, and usefulness of monitoring data
	Partnership/collaboration with external agencies	Formal and informal working arrangements with other agencies
Staff/case worker	Buy‐in (commitment to programme)	Understanding and support from delivery (implementation) level staff/case workers
	Communication and engagement with programme recipient	De facto and de jure arrangements for and occurrence of communication with programme recipients by staff/case workers
	Communication and engagement with other agencies	De facto and de jure arrangements for and occurrence of communication with other agencies by staff/case workers
	Emotional skills (Awareness, building trust, taking a personalized approach)	Level of emotional intelligence and skill displayed by staff/case workers
	Technical skills (capabilities, training)	Technical capacity of staff/case workers to perform their jobs, and support for that capacity
Recipient of programme	Buy‐in (emotional acceptance of programme)	Acceptance of the support offered by the project or programme by intended recipients
	Access to non‐housing support (medical, financial, training etc.)	Access to non‐housing support services necessary for programme implementation to be successful
	Housing‐related security	Provision to stay in appropriate housing to prevent a recurrence of homelessness
	Adequacy of information provided	The quantity and quality of the information provided about the programme to intended beneficiaries
	Accessibility (time and place)	Accessibility of the services provided by the programme in terms of time and space

### 3.2 Criteria for including and excluding studies

#### 3.2.1 Types of study designs

This is a map of the implementation issues of interventions to improve the welfare of those experiencing, or at risk of, homelessness. The map will include process evaluations and other studies of barriers and facilitators for eligible interventions, systematic reviews of such studies, and mixed method studies.

For primary studies, We are including a broad range of all qualitative study designs because implementation issues are examined in a variety of different ways across the included interventions, and we want to capture all studies examining barriers and facilitators of implementation. We will include any quantitative study that includes measures of implementation. These may be outcome studies that also quantitatively measured and reported implementation barriers and facilitators. Mixed methods studies will also be included. There may be studies that used a quantitative method to examine outcomes of homeless interventions, and a qualitative method to assess implementation. Studies with quantitative study design alone that do not report implementation issues will be excluded. There is a separate EGM for the effectiveness of interventions for those experiencing or likely to experience homelessness.

To qualify as a systematic review, the study must (1) have a search strategy with explicit inclusion criteria, (2) search at least two databases, (3) systematically code, analyze and report on all included studies.

#### 3.2.2 Types of settings

Studies will be from high‐income countries.

### 3.3 Search strategy and status of studies

We will search several databases, websites and registries, conduct backward citation tracking of included studies, contact researchers and hand search selected journals to search for published and unpublished studies. Many process evaluations are less likely to be found in academic databases. The grey literature search is thus likely to retrieve process evaluations. In addition to these, we will also do hand searches of selected journals.

The EGM will include studies published in English language and is not confined to any specific time period.

#### 3.3.1 Database search

The databases to be searched will be searched as part of the effectiveness map in which screening will identify studies suitable for the implementation issues map. That search includes:

1. Academic databases
  – Econlit
  – The National Bureau of Economic Research (NBER)
  – Social Science Research Network (SSRN)
  – International Bibliography of Social Sciences (IBSS)
  – Applied Social Sciences Index and Abstracts (ASSIA)
  – Social Service Abstract
  – Embase
  – PubMed
  – PsychINFO
  – MEDLINE
  – WHO Global Index Medicus
  – CABI's Global Health
  – ERIC
  – CINAHL
  – SCOPUS
  – Science Citation Index and Social Sciences Citation Index files from Web of Science
  – EPPI Centre Evaluation Database of Education Research
  – Social Policy and Practice
  – Proquest Theses and Dissertations Global
2. EGM database
  – 3ie EGM repository
  – Global Evidence Mapping Initiative
  – Evidence based Synthesis Programme (Department of Veteran affairs)
3. Systematic review databases
  – International Health Technology Assessment Database
  – Collaboration for Environmental Evidence
  – Cochrane Database of Systematic reviews
  – Campbell Systematic Reviews
  – 3ie Systematic Review Database
  – Research for Development
  – Epistemonikos

Sample search terms are listed in Supporting Information: Appendix 1.

#### 3.3.2 Grey literature and websites

In addition to electronic studies, we shall search and screen publications from the following websites.

We will search for studies in the US, UK, Australia, Canada and New Zealand by searching websites for states or provinces (and counties in the UK) through a search of their website. We will also use search engines like Google in the incognito mode to search for evaluations on homelessness by using key words like ‘homelessness evaluation’ AND the name of the country. For example, to search homelessness evaluations in Australia, we will use the search term ‘homelessness evaluation’ AND Australia. We will also apply the same approach for major cities in specific countries.

We will also use various synonyms of interventions (while using Boolean operator OR) and combining them with various synonyms for studies with implementation issues (using Boolean operator AND) in Google to identify eligible studies. For example;

(Effectiveness OR impact evaluation OR Implementation OR Barriers and facilitators OR Process Evaluation OR Evaluation) AND (Outreach access and recover OR assertive outreach OR street team OR multidisciplinary street team OR intensive outreach OR community prevention)^2^.

The list of websites to be searched is as follows:

Homeless Hub https://www.homelesshub.ca/

European observatory on homelessness https://www.feantsaresearch.org/en/publications

United State interagency council on homelessness http://www.usich.gov/

EThOS http://ethos.bl.uk/Home.do

WHO ICTRP http://apps.who.int/trialsearch/

Focus on Prevention http://www.preventionfocus.net/

100,00 home campaigns https://en.wikipedia.org/wiki/100,000_Homes_Campaign

Anti poverty committee https://en.wikipedia.org/wiki/Anti-Poverty_Committee

Back on my feet https://en.wikipedia.org/wiki/Back_on_My_Feet_(non‐profit_organization)

Feantsa https://www.feantsa.org/

National Coalition Homeless https://nationalhomeless.org/

Homelessness Australia https://www.homelessnessaustralia.org.au/

Mission Australia https://www.missionaustralia.com.au/publications/position-statements/homelessness

National Alliance to end homelessness https://endhomelessness.org/

Institute of global homelessness https://www.ighomelessness.org/

Homelessness link https://www.homeless.org.uk/

Crisis https://www.crisis.org.uk/about-us/how-we-work/

Housing first https://housingfirsteurope.eu/about-the-hub/

Canadian Alliance to end homelessness https://housingfirsteurope.eu/about-the-hub/

Social work and policy institutes http://www.socialworkpolicy.org/research/homelessness.html

Association of housing advice services https://www.ahas.org.uk/

Centre point https://centrepoint.org.uk/

Homelessness trust funds https://housingtrustfundproject.org/htf-elements/homeless-trust-funds/

Meliville charitable trust https://melvilletrust.org/category/resources-reports/

Conrad H Hilton foundation https://www.hiltonfoundation.org/priorities/homelessness#resources

Abt Associates https://www.abtassociates.com/

Mathematica https://www.mathematica-mpr.com/

American Institutes of Research https://www.air.org/

Rand https://www.rand.org/

MDRC https://www.mdrc.org/

#### 3.3.3 Contacting researchers

We will send copies of the preliminary map to authors of included studies, which serves both a dissemination purpose and to invite submission of additional studies.

#### 3.3.4 Hand searches of selected journals

These journals were selected based on Google scholar searches identified by using keywords like ‘homelessness evaluation journals’, ‘housing policy journals’ and by scanning through the journal titles obtained from database searches. The handsearches of journals ensure that any reports that may not feature in database searches at a given point in time due to any delays in indexing are also covered.

*European Journal of Homelessness*

*Health & Social Care in the Community*

*Housing Care and Support*

*International Journal of Housing Policy*

*International Journal on Homelessness*

*Journal of Social Distress and the Homeless*

*Parity*

##### Screening of the studies

For the data management and both stages of screening and data extraction, Eppi Reviewer 4 and Web version of Eppi Reviewer will be used. The screening at title and abstract will be done by two researchers independent of each other. The disagreements will be resolved by discussion or by approaching an arbitrator. The full‐text screening will also be done by two researchers independent of each other. The same procedure will be used as at the title and abstract screening for resolving disagreements. The data will be extracted from the studies found eligible at the full‐text screening stage.

### 3.4 Data extraction, coding and management

Coding will be done independently by two coders, with a third party arbitrator in the event of disagreement.

#### 3.4.1 Coding of bibliographic information and intervention and study design and characteristics

Full bibliographic information will be captured, along with the information necessary to construct the map (interventions, implementation issues and filters). The coding form is given in Supporting Information: Appendix 2.

#### 3.4.2 Critical appraisal

Coding will also capture the data needed for critical appraisal of all included studies. The confidence in findings of included systematic reviews will be assessed using AMSTAR 2. Critical appraisal of primary studies shall be conducted using the tool contained in Supporting Information: Appendix 3. This tool is referred to as the Keenan‐White (KW) tool and is developed for the use in the homelessness implementation map. The KW tool is based on three existing critical appraisal tools: CASP, SURE, and JBI. The motivation for KW was to separate out items which were conflated in existing tools, to use plain language, and stick to items for which the responses are most likely replicable between coders. In selecting questions to go in the critical appraisal tool for this map, we aimed to create a tool to appraise ‘confidence in study findings’. The tool was modified through piloting with numerous studies to ensure studies could be effectively rated on the scale.

The overall rating in the tool is based on a modification to the ‘weakest link in the chain’ principle, that is, the overall rating equals the lowest rating on any critical item.

As at the screening and data extraction stages, the critical appraisal is also done by two coders independently. The responses to various items are then matched for comparison. The disagreements are then resolved by discussion. An arbiter reconciles if disagreement persists after discussion between the coders.

### 3.5 Analysis and presentation

#### 3.5.1 Unit of analyses

The unit of analysis is a study with multiple reports. We will compare the reports for a single study and the norm is that most recent and comprehensive study with more complete and detailed information is represented on the map. This is specifically apt for a protocol and completed study findings report where the report gets published after a protocol (latest) and it also has the study findings (comprehensive).

All reports of the same study with more or less same data are linked in the EPPI Reviewer (software) but the latest and comprehensive one is depicted on the map. If any of the reports have different analyses for a single study, they are plotted separately on the map. Hence, in principle, there may be multiple entries from a single study. If any study accounts for more than 10 papers or reports that study shall be included as a filter. The accompanying EGM report will identify the number of studies covered by the map and list those studies with multiple papers in an appendix.

#### 3.5.2 Presentation

The intervention and implementation issues, described above, are the primary dimensions of the map.

In addition to intervention and outcomes, the following filters will be coded for primary studies (and reviews where appropriate):

1. Global Region (names of regions)
2. Country
3. National region (e.g., state in the US, or country in UK such England)

#### 3.5.3 Planned analyses

The EGM report shall provide tabulations or graphs of the number of studies, with accompanying narrative description, by

- Intervention category and sub‐category
- Implementation issue domain and sub‐domain
- Table of ‘aggregate map’ of interventions and barriers
- Table of ‘aggregate map’ of interventions and facilitators
- Region and country
- Year
- Study type

The narrative description will also include some examples from certain studies included in the map, that is, barriers and facilitators identified in a specific study will be given to provide some contextual information to the reader.

### 3.6 Stakeholder engagement

The framework was developed through a consultative process. The intervention framework was developed as follows:

1. Two existing frameworks were considered as a basis for the framework to be used for this map: (1) the intervention categories used by Munthe‐Kaas et al. (2018), and (2) the categories provided by Crisis (which are used in the SCIE, 2018; review).
2. The proposed framework was reviewed by staff of Crisis and a group of UK academics specializing in homelessness (I‐SPHERE) and revised on the basis of their comments and further discussion with the Director of the new What Works Centre for Homelessness.
3. A group of homelessness researchers and practitioners reviewed the categories in an interactive exercise to fit the identified papers into the categories, resulting in further revision of those categories.
4. The framework was further revised after 18 months of use of the first version of the map.

The map will be discussed with the Advisory Group for the Centre for Homeless Impact and presented at consultations organized by the Centre.

## CONTRIBUTIONS OF AUTHORS

**Lead EGM author**: The lead author is the person who develops and co‐ordinates the EGM team, discusses and assigns roles for individual members of the map team, liaises with the editorial base and takes responsibility for the on‐going updates of the map.

### 1 ROLES AND RESPONSIBILITIES

Content: Sabina Singh, Ting Yang, Ligia Teixeira, Suzanne Fitzpatrick and Jenny Wood. Sabina Singh is Director Research at Campbell South Asia, Global development Network. She is a social anthropologist by training and has led systematic reviews and several EGMs. Ting Yang is an evaluator specializing in evaluation methods and impact evaluation with a focus on qualitative methods. Ligia Teixeira is Director of the new UK CHI. Professor Suzanne Fitzpatrick has been researching homelessness for two decades with many scientific and official publications on the topic, and is a former editor of the *International Journal of Housing Policy*. Jenny Wood is a Research Associate at the Institute for Social Policy, Housing and Equalities Research at Heriot‐Watt University.

Evidence gap methods: Howard White and Ashrita Saran, who have co‐authored a paper on mapping methods used by different agencies. Howard White assisted development of Campbell guidelines and standards for EGMs. They are authors of the homelessness effectiveness map.

Information retrieval: Ashrita Saran and Howard White. Ashrita Saran has received training on search strategies and authored strategies for other evidence synthesis products. The search strategy was adopted from that used by Munthe‐Kaas et al. (2018). The strategy was reviewed by John Eyres (IDCG Search Specialist) before submission. Search methods other than database searches will be carried out by teams led by Sabina Singh and Monisha Lakshminarayanan. Monisha Lakshminarayanan is Lead Evidence Synthesis Specialist at Campbell South Asia.

Project management: Howard Whitewill manage the project to ensure timely delivery.

## DECLARATIONS OF INTEREST

Ligia Teixeira is Director of the Centre for Homelessness Impact. This role should not provide any conflict as CHI's mission is to make evidence available. Suzanne Fitzpatrick is a leading researcher in the area so some her studies may be eligible for inclusion in the map. Howard White was CEO of the Campbell Collaboration; he has no role in the editorial process for studies published by the Campbell Collaboration. He is presently Director Evaluation and Evidence Synthesis at Global Development Network, New Delhi.

### 1 PLANS FOR UPDATING THE EGM

The Centre for Homelessness Impact has agreed to provide resources to update the map every two years. The EGM team are in discussions with the EPPI Centre, who are responsible for the mapping software, about possible real time updating through (1) automated searches with machine‐learning powered screening, and (2) moderated submissions of suggested papers.

## SOURCES OF SUPPORT

### 1 Internal sources

- Production of the map has been supported by the UK Centre for Homelessness Impact, with in‐kind support from the Campbell Collaboration Secretariat, Other.

### 2 External sources

- No sources of support provided

## Supporting information

Supporting information.

## Footnotes

^1^

https://www.bigissue.com/latest/social-activism/how-many-people-are-homeless-in-the-uk-and-what-can-you-do-about-it/#:~:text=Overall%2C%20Shelter%20estimated%20that%20280%2C000,councils%20for%20help%20with%20homelessness

^2^
The database searches as well as grey literature searches for effectiveness and implementation maps of homelessness will be carried out simultaneously and the studies for both maps will be separated at the beginning. Any discrepancies will be taken care at the screening stage itself.
