# Supplementary material for: PROTOCOL: An evidence and gap map of studies of implementation issues for interventions for those affected by and at risk of homelessness in high‐income countries
Source: Campbell Syst Rev. 2023 Feb 21;19(1):e1305. doi: 10.1002/cl2.1305 (PMC9942265; doi:10.1002/cl2.1305)
Supplement: Supplementary file 1 — Supporting information. [file CL2-19-e1305-s001.docx]

# Appendices

## 1 Appendix 1 Sample Search String

Search string/Key words (For Ovid Medline platform)

-

Study design key words

- homeless evaluation

- evaluation

- homeless/ness

Homelessness key words

- homeless persons/ or homeless youth/

- (evict* or homeless* or "housing excl*" or "residential stability" or ((street* or private or improvised or shelter* or emergency or temporary or insecure or overcrowded or precarious or stable or marginal*) adj3 (dwell* or house* or housing or accommodation)) or (street adj3 (life or living or lives or youth* or child* or people or person*)) or runaway* or "Run away from home" or "Running away" or "Ran away" or "Going missing" or "Bag lady" or Houseless* or Unhoused or "without a roof" or Roofless or (rough adj3 sleep*) or Destitut* or "Skid row*" or "sleepers out").ti,ab,kw.

- ("Housing first" or "Pathways to Housing" or "Homeless Veterans Reintegration Program" or "Access to Community Care and Effective Services and Supports" or "Support* Housing Program" or "Housing and Urban Development–Veterans Affairs Supported Housing program" or "HUD-VASH" or "Sober Transitional Housing and Employment Project" or "sober house placement*" or "Housing ladders" or "Staircase housing" or "low threshold housing" or "Critical Time Intervention").ti,ab,kw.

## 2 Appendix 2 Key items from coding form

- Region
  - Australasia
  - Western Europe
  - North America
- Country
  - Austria
  - Australia
  - Belgium
  - Canada
  - Czech Republic
  - Denmark
  - England
  - France
  - Finland
  - Germany
  - Hungary
  - Ireland
  - Italy
  - Japan
  - Netherlands
  - New Zealand
  - Norway
  - Poland
  - Portugal
  - Scotland
  - Spain
  - Sweden
  - United Kingdom
  - United States
- Place
  - Not reported
  - Aberdeen
  - Alabama
  - Albany
  - Alberta
  - Albuquerque
  - Amsterdam
  - Anchorage
  - Arlington County
  - Arizona
  - Auckland
  - Austin
  - Baltimore
  - Bologna
  - Barcelona
  - Barnardos
  - Bayside Peninsula
  - Belfast
  - Bellingham
  - Bergen
  - Bradford
  - Brighton
  - Birkenhead
  - Brimingham
  - Brisbane
  - Bristol
  - British Columbia
  - Boston
  - Budapest
  - Buffalo
  - Brussels
  - Cambridgeshire
  - Camperdown
  - Cardiff
  - Catalonia
  - Chermsland
  - Cairns
  - California
  - Calgary
  - Chicago
  - Cincinnati
  - Colarado
  - Colombus
  - Columbia
  - Connecticut
  - Contra Costa
  - Copenhagen
  - Cork
  - Cornwall
  - Costa County
  - Creighton
  - Darwen
  - Dayton
  - Decatur
  - Delaware
  - Denver
  - Drammen
  - Dublin
  - East Surrey
  - Edinburgh
  - England
  - Espoo
  - Essex
  - Fall River
  - Florida
  - Fremantle
  - Gateshead
  - Georgia
  - Ghent
  - Girona
  - Glasgow
  - Gloucestershire
  - Gold Coast
  - Grafton
  - Greater Manchester
  - Greater Victoria
  - Great Yarmouth
  - Halton (Runcorn and Widnes)
  - Halifax Regional Municipality
  - Hannibal
  - Helsinki
  - Houston
  - Illinois
  - Indiana
  - Ipswich
  - Irns
  - Jefferson
  - Joensuu
  - Jyväskylä
  - Kentucky
  - Kimberley
  - Kununurra
  - Kuopio
  - Lancaster
  - Lahti
  - Lane County
  - Leeds
  - Leicester
  - Lille
  - Lincolnshire
  - Lisbon
  - Lismore
  - Liverpool
  - Loathian
  - London
  - Los Angeles
  - Louisiana
  - Luton
  - Madrid
  - Malaga
  - Mallee
  - Maine
  - Manchester
  - Manitoba
  - Maricopa
  - Marseille
  - Marsham
  - Maryland
  - Massachusetts
  - Melbourne
  - Merseyside
  - Mexico
  - Miami
  - Michigan
  - Minnesota
  - Missisauga
  - Missouri
  - Moncton
  - Montana
  - Montgomery County
  - Montréal
  - Mt. Isa
  - Nepean
  - New Brunswick
  - New Castle
  - New Hampshire
  - New Jersey
  - New Orleans
  - New South Wales
  - New South Wales
  - New York
  - Nijmegen
  - Norfolk
  - North Carolina
  - North Yorkshire
  - Nottingham
  - Norwich
  - Oakland
  - Ohio
  - Oldham
  - Ontario
  - Orlando
  - Ottawa
  - Oulu
  - Overland Park
  - Oxford
  - pasco county
  - Paris
  - Peninsula
  - Pennsylvania
  - Perth
  - Phoenix
  - Peterborough
  - Philadelphia
  - Pima County
  - Pittsburgh
  - Plymouth
  - Portland
  - Quebec
  - Queensland
  - Queensland
  - Rockhampton
  - Rhode Island
  - Rimini
  - Rome
  - Ryedale
  - Sacramento
  - San diego
  - San francisco
  - Santa Clara
  - Santa Monica
  - Sault Ste. Marie
  - Scotland
  - Seattle
  - Sheffield
  - Siracusa
  - Springfield
  - Stoke-on-Trent
  - South Australia
  - South Jordan
  - Southend-on-Sea
  - Southwark
  - South Carolina
  - South Yorkshire
  - Stockport
  - Suffolk Coastal
  - Surrey
  - Sussex
  - Sydney
  - Tameside
  - Tampere
  - Tasmania
  - Texas
  - Tollington Way
  - Tokyo
  - Toronto
  - Toulouse
  - Townsville
  - Trenton
  - Turku
  - Tweed Heads
  - Utah
  - Vancouver
  - Vantaa
  - Verona
  - Victoria
  - Vienna
  - Vinnies
  - Virginia
  - Volker
  - Waterloo
  - Wales
  - Washington D.C
  - Waterford
  - Western Australia
  - West Lothian
  - West Moreton region
  - Weston-super-Mare
  - West Yorkshire
  - Wellington
  - Winnipeg
  - Wisconsin
  - Wolverhampton
  - Worcester
  - Wyndham
  - York
  - Ynys Môn (The Isle of Anglesey)
- Interventions
  - Legislation
    - Housing/Homelessness legislation
    - Welfare benefits
    - Health and social care
  - Prevention
    - Welfare and housing support
    - Housing supply
    - Family therapy and mediation
    - Landlord tenant mediation
    - Discharge
  - Services and outreach
    - Feeding
    - In kind support
    - Day centres
    - Outreach
    - Reconnection
    - Psychologically informed environments
    - Case management / Critical time intervention
    - Service coordination
    - Veterinary services
    - Legal advice
  - Accommodation based interventions
    - Shelters
    - Hostels
    - Temporary accommodation
    - Host homes
    - Rapid rehousing
    - Housing first
    - Social housing
    - Private rented sector (with and without support)
    - Continuum of Care
  - Employment
    - Mentoring and coaching
    - Flexible employment
    - Vocational training and unpaid work experiences
    - Paid work experiences
  - Health and social care
    - Health services
    - End of life
    - Addiction support
  - Education and skills
    - Vocational training
    - Work experience
    - Life skill training
    - Education
    - Creative activities
  - Communication
    - Advocacy campaigns
    - Public information campaigns
    - Service availability
  - Financing
    - Social Impact Bonds
    - Direct financial support from public
- Barriers
  - B1. Contextual factors
    - B1.1 Housing market
    - B1.2 Labour Market
    - B 1.3 Welfare support
    - B 1.4 Law
  - B2. Policy makers/funders
    - B 2.1 Buy-in (Leadership, culture, priorities, commitment to programme)
    - B 2.2 Contracting arrangements with external agencies
    - B 2.3 Framework provision (e.g. policies and guidelines)
  - B3. Program administrator/ manager/ implementing agency
    - B 3.1 Buy in (Leadership, culture, priorities)
    - B 3.2 Identification of recipient /targeting mechanism
    - B 3.3 Referral route (e.g. defined agency or contact)
    - B 3.4 Sufficiency/Adequacy of Resources (space, time, staff, budget
    - B 3.5 Alignment with existing protocol/ procedures/ guidelines
    - B 3.6 Monitoring data/Data sharing
    - B 3.7 Partnership/collaboration with external agencies
  - B4. Staff/ case worker
    - B 4.1 Buy-in (commitment to program)
    - B 4.2 Communication and engagement with programme recipient
    - B 4.3 Communication and engagement with other agencies
    - B 4.4 Emotional skills (Awareness, empathy, building trust, taking a personalised approach)
    - B 4.5 Technical skills (capabilities, training)
  - B5. Recipient of program
    - B 5.1 Buy-in (emotional acceptance of program)
    - B 5.2 Access to non-housing support (medical, financial, training etc.)
    - B 5.3 Housing-related security
    - B 5.4 Adequacy of information provided
    - B 5.5 Accessibility (time and place)
- Facilitators
  - F1. Contextual factors
    - F 1.1 Housing Market
    - F 1.2 Labour Market
    - F 1.3 Welfare Support
    - F 1.4 Law
  - F2. Policy maker/ funders
    - F 2.1 Buy in (Leadership, culture, priorities, commitment to programme
    - F 2.2 Contracting arrangements with external agencies
    - F 2.3 Framework provision (e.g. policies and guidelines
  - F3. Program administrator/ manager/ implementing agency
    - F 3.1 Buy in (Leadership, culture, priorities
    - F 3.2 Identification of recipient /targeting mechanism
    - F 3.3 Referral route (e.g. defined agency or contact)
    - F 3.4 Sufficiency/Adequacy of Resources (space, time, staff, budget, appropriateness of services or facilities)
    - F 3.5 Alignment with existing protocol/ procedures/ guidelines
    - F 3.6 Monitoring data/Data sharing
    - F 3.7 Partnership/collaboration with external agencies
  - F4. Staff/ case worker
    - F 4.1 Buy-in (commitment to program)
    - F 4.2 Communication and engagement with programme recipient
    - F 4.3 Communication and engagement with other agencies
    - F 4.4 Emotional skills (Awareness, empathy, building trust, taking a personalised approach)
    - F 4.5 Technical skills (capabilities, training)
  - F5. Recipient of program
    - F 5.1 Buy-in (emotional acceptance of program)
    - F 5.2 Access to non-housing support (medical, financial, training etc.)
    - F 5.3Housing-related security
    - F 5.4 Adequacy of information provided
    - F 5.5 Accessibility (time and place)
- B&F-TYPE
  - Barriers
  - Facilitators
  - Ongoing
- Barriers and Facilitators
  - BF 1. Contextual factors
    - BF 1.1 Housing market
    - BF 1.2 Labour Market
    - BF 1.3 Welfare support
    - BF 1.4 Law
  - BF 2. Policy makers/funders
    - BF 2.1 Buy-in (Leadership, culture, priorities, commitment to programme)
    - BF 2.2 Contracting arrangements with external agencies
    - BF 2.3 Framework provision (e.g. policies and guidelines)
  - BF 3. Program administrator/ manager/ implementing agency
    - BF 3.1 Buy in (Leadership, culture, priorities)
    - BF 3.2 Identification of recipient /targeting mechanism
    - BF 3.3 Referral route (e.g. defined agency or contact)
    - BF 3.4 Sufficiency/Adequacy of Resources (space, time, staff, budget
    - BF 3.5 Alignment with existing protocol/ procedures/ guidelines
    - BF 3.6 Monitoring data/Data sharing
    - BF 3.7 Partnership/collaboration with external agencies
  - BF 4. Staff/ case worker
    - BF 4.1 Buy-in (commitment to program)
    - BF 4.2 Communication and engagement with programme recipient
    - BF 4.3 Communication and engagement with other agencies
    - BF 4.4 Emotional skills (Awareness, empathy, building trust, taking a personalised approach)
    - BF 4.5 Technical skills (capabilities, training)
  - BF 5. Recipient of program
    - BF 5.1 Buy-in (emotional acceptance of program)
    - BF 5.2 Access to non-housing support (medical, financial, training etc.)
    - BF 5.3 Housing-related security
    - BF 5.4 Adequacy of information provided
    - BF 5.5 Accessibility (time and place)
- Critical appraisal
  - Are the research questions clearly stated?
    - Yes
    - Partly
    - No
  - Is the qualitative methodology adequately described?
    - Yes
    - Partly
    - No
  - Is the qualitatively methodology appropriate?
    - Yes
    - Partly
    - No
  - Is the recruitment/sampling strategy adequately described?
    - Yes
    - Partly
    - No
  - Is the recruitment/sampling strategy appropriate?
    - Yes
    - Partly
    - No
  - Relationship between researchers and participants
    - Yes
    - Partly
    - No
  - Ethical considerations
    - Yes
    - Partly
    - No
    - Insufficient detail
  - Is the data collection approach adequately described?
    - Yes
    - Partly
    - Insufficient detail
  - Is the data analysis approach adequately described?
    - Yes
    - Partly
    - No
  - Is the data analysis sufficiently rigorous?
    - Yes
    - Partly
    - No
  - Clear statement of policy recommendations
    - Yes
    - Partly
    - No
  - Recommendations based on the report findings
    - Yes
    - Partly
    - No
  - Overall
    - Low
    - Medium
    - High
- Confidence in study findings
  - Low
  - Medium
  - High
- Type of Implementation issues
  - Barriers
  - Facilitators

## 3 Appendix 3 Critical appraisal tool for primary studies (qualitative)

| **Qualitative critical appraisal tool** | |  |  |  |  |  |  |  |  |
| --- | --- | --- | --- | --- | --- | --- | --- | --- | --- |
|  |  |  | Response |  |  |  |  |  |  |
|  |  |  |  |  |  |  |  |  |  |
| 1 | Are the research questions clearly stated? |  | Yes |  | Partly |  | No |  |  |
|  |  |  |  |  |  |  |  |  |  |
| 2 | Is the qualitative methodology adequately described? |  | Yes |  | Partly |  | No | >> 4 |  |
|  |  |  |  |  |  |  |  |  |  |
| 3 | Is the qualitatively methodology apprpriate to address the research questions? |  | Yes |  | Partly |  | No |  |  |
|  |  |  |  |  |  |  |  |  |  |
| 4 | Is the recruitment/sampling strategy adequately described? |  | Yes |  | Partly |  | No | >> 6 |  |
|  |  |  |  |  |  |  |  |  |  |
| 5 | Is the recruitment/sampling strategy appropriate to address the research questions? |  | Yes |  | Partly |  | No |  |  |
|  |  |  |  |  |  |  |  |  |  |
| 6 | Has the relationship between researchers and participants been adequately considered? |  | Yes |  | Partly |  | No |  |  |
|  |  |  |  |  |  |  |  |  |  |
| 7 | Have ethical considerations been sufficiently considered? |  | Yes |  | Partly |  | No |  | Insufficient detail |
|  |  |  |  |  |  |  |  |  |  |
| 8 | Is the data collection approach adequately described? |  | Yes |  | Partly |  | No |  | Insufficient detail |
|  |  |  |  |  |  |  |  |  |  |
| 9 | Is the data analysis approach adequately described? |  | Yes |  | Partly |  | No | >>11 |  |
|  |  |  |  |  |  |  |  |  |  |
| 10 | Is the data analysis sufficiently rigorous? |  | Yes |  | Partly |  | No |  |  |
|  |  |  |  |  |  |  |  |  |  |
| 11 | Is there a clear statement of policy recommendations or implications of the research? |  | Yes |  | Partly |  | No |  |  |
|  |  |  |  |  |  |  |  |  |  |
| 12 | Are the findings or recommendations based on the report findings? |  | Yes |  | Partly |  | No |  |  |
